# 

**Published:** 1900-04

**Authors:** 


					APRIL, 1900.
THE
AMERICAN JOURNAL
O F
DENTAL SCIENCE.
EDITMfBX:
F. J. S. GORGES, M. D, D. D. S.
RICHARD GRADX^--?Ctr^r "s7
Vol. 33.
THIRD SERIES
S o. 1?.
BALTIMORE:
SNOWDEN 4. COWMAN MFG. CO., 9 W. Fayette St.
LONDON:
TRUBNER &. CO., 60 Paternoster Row.
Entered at the Post Office at Baltimore, Md., as Second-class Matter.
PSYKBLE IN ^DV^NCE.
PUBLISHED MONTHLY.
$2.b0 PER HNNUM.I

				

